# The Legal Determinants of Health: How Can We Achieve Universal Health Coverage and What Does it Mean?

**DOI:** 10.34172/ijhpm.2020.64

**Published:** 2020-05-11

**Authors:** Lawrence O. Gostin

**Affiliations:** O’Neill Institute for National and Global Health Law, Georgetown University Law Center, Washington, DC, USA.

**Keywords:** Universal Health Coverage, Global Health, Public Health, Equity, Justice

## Abstract

How can we keep people – wherever they live – healthy and safe? Among all global health initiatives, universal health coverage (UHC) has garnered most political attention. But can UHC (as important as it is) actually achieve the two fundamental aspirations of the right to health: keeping people healthy and safe, while leaving no one behind? There is a universal longing for health and security, but also a deep-seated belief in fairness and equity. Can UHC achieve both health and equity, or what I have called, "global health with justice?" What makes a population healthy and safe? Certainly, universal and affordable access to healthcare is essential, including clinical prevention, treatment, and essential medicines. But beyond medical care are public health services, including surveillance, clean air, potable water, sanitation, vector control, and tobacco control. The final and most important factor in good health are social determinants, including housing, employment, education, and equity. If we can provide everyone with these three essential conditions for good health (healthcare, public health and social determinants), it would vastly improve global health. But we also need to take measures to leave no one behind. To achieve equity, we need to plan for it, and here I propose national health equity programs of action. Society’s highest obligation is to achieve global health, with justice.

How can we keep people – wherever they live – healthy and safe? Among all global health initiatives (eg, Eradicate Polio, Roll Back Malaria, Stop TB), universal health coverage (UHC) has garnered most political attention. But can UHC (as important as it is) actually achieve the two fundamental aspirations of the right to health: keeping people healthy and safe, while leaving no one behind?^1^ There is a universal longing for health and security, but also a deep-seated belief in fairness and equity. Can UHC achieve both health and equity, or what I have called, “global health with justice?”^2^ I will return to this all-important question, but first let’s explore the expansive political support for UHC as well as the meaning of that term.

## The Political Impact and Meanings of Universal Health Coverage

The idea of UHC is very much in political fashion. The Sustainable Development Goals (SDGs), adopted by all United Nations (UN) Member States in 2015, have a single health goal, “Ensure healthy lives and promote well-being for all at all ages.”^3^ Its most important target is to achieve UHC by 2030. World Health Organization (WHO) Director-General Tedros Adhanom, declaring, “All roads lead to UHC,” has made universal coverage WHO’s highest priority.^4^ And last October, the UN General Assembly unanimously adopted a historic political declaration, “UHC: Moving Together to Build a Healthier World.”^5,6^ Such political commitment is essential, for meeting the SDG target on UHC is as ambitious as it is imperative: as of 2017, less than half of the world’s population had access to essential health services.^7^

Strong and resilient health systems are vital for health, but what exactly does the international community mean by the ideal of UHC? In fact, UHC definitions vary widely, which is troubling. The UN, WHO, and World Bank all stress financial risk protection, that is, healthcare costs should not push people into poverty. That is a worthwhile goal, yet definitions of UHC are actually quite limited. Even as other aspects of the SDGs encompass a range of public health functions, the SDGs limit UHC itself solely to medical and nursing care: “access to quality essential healthcare services and access to safe, effective, quality and affordable essential medicines and vaccines for all.”^8^ The World Bank’s UHC definition also stresses healthcare, noting that health services support nations’ strongest asset: human capital, a foundational investment in economic growth.^9^

The WHO has a broader concept of UHC, embracing prevention as well as treatment, with all people able to “use the promotive, preventive, curative, rehabilitative and palliative health services they needs.”^10^ Importantly, WHO and the Bank jointly monitor UHC implementation, using WHO’s definition, including sanitation, non-communicable diseases (eg, diabetes, heart disease, tobacco control) and health security (preparedness for fast-moving epidemic diseases).^11^ This more expansive concept of UHC is welcome, but WHO rarely advocates for population-based public health services as part of the package of UHC services. That is, WHO – in its policies, advocacy, and expenditures – do noes not strongly focus on a strong public health infrastructure.

## What Makes a Population Healthy and Safe?

If the ultimate aim of UHC is to achieve healthier, safer populations, then what are the key determinants of health and well-being? Healthcare, of course, is important. Everyone wants affordable access to diagnostic, treatment, and rehabilitative services, including emergency and palliative care services. Yet, medical care constitutes only a small proportion of what makes a population healthy. More important are public health services, encompassing clean air, potable water, vector control, injury prevention, and tobacco and alcohol control. People thrive if they live, work, and play in healthy environments that encourage physical activity (walking, biking, recreating) and a nutritious diet (fresh fruits, vegetables, lean protein). In sum, the environment in which we live makes all the difference to our health. Our natural and physical environments must be conducive to health. Our built environment must be structured so that health is the “easier choice.”

And as important as healthcare and public health are, the truth is that the single greatest influence on people’s health are the services and opportunities that reside well outside the health sector. The social determinants of health include income, education, housing, social support, and gender/racial equality.^12^ That is why health requires an “All-of-Government” approach, where the full range of ministries take health fully into account in their policies, practices, and funding.

## Why Is Justice Good for Your Health?

Health is determined not only by the services people can access and the environments in which they live. Health also requires equity. Societies that have large disparities in income, education, and social status also tend to have less healthy populations overall. Consider the experience in the United States, where life expectancy has fallen for the last three years, after steady progress for decades.^12^ Most of the loss of life is caused by so-called “diseases of despair”—alcohol and drug (opioid) dependencies, depression, and suicides. Further, a disproportionate burden of premature mortality is falling on the poor and middle class, who have fallen further behind while the rich get ever richer.

It is for this reason that all countries should adopt, fund, and rigorously implement national health equity programs of action– systematic, systemic, and inclusive approaches to achieve health equity. An international group of scholars and advocates identified seven key principles for health equity programs of action.^13^ Programs of action should be developed through inclusive, participatory, empowering processes; have the express goal of maximizing health equity; encompass both the health sector and other sectors, including the full range of social, environmental, economic, commercial, and political determinants of health; comprehensively identify all populations experiencing health inequities, analyzing their particular obstacles to good health and identifying actions to overcome them; be action-oriented, with specific targets and timelines; include measures to ensure accountability; and be backed by sustained high-level political commitment, with leadership from heads of government. The only way to significantly close the health disparity divide is to measure who is left behind and why, and to take concrete action to promote health equity.

## The Legal Determinants of Health

A 2019 Lancet Commission on Global Health and the Law coined the term “the legal determinants of health” to show how law can be a powerful tool for ensuring the public’s health and safety. This tool must be used to promote health and rights. For law can also pose an obstacle to good health, such as by criminal laws targeting persons living with HIV/AIDS, laws limited sexual and reproductive health services, and criminalization of LGBT (lesbian, gay, bisexual, and transgender) population.^14^

Whatever the definition, UHC can be accomplished only through the law. At the September 2019 UN General Assembly, WHO, United Nations Development Programme, the Joint United Nations Programme on HIV/AIDS (UNAIDS), the Inter-Parliamentary Union, and the O’Neill Institute at Georgetown University launched the Legal Solutions for UHC Network to support national law reform.^15^ There are three core legal determinants of health needed to achieve UHC: (1) health laws must fulfill each core element of UHC; (2) health systems must be well-governed; and (3) public officials must abide by the rule of law.^14^

Advancing the right to health through UHC requires adherence to five key values. Health services must be universally accessible, equitable, affordable, of high quality, and cost effective. A comprehensive national health law should ensure that everyone in the country is eligible for the full package of health services, medicines and vaccines. No one should be excluded irrespective of their income, gender, race, legal residence, or other status. In many countries, coverage of unlawful residents and migrants is most controversial, and most governments do not extend full (or even any) coverage to these groups.^16^ Yet, exclusion of migrants from full access to the health system is guaranteed to undermine the SDG target of UHC.^17^ Furthermore, there should not be special eligibility criteria for health coverage, such as work requirements.

The next value of a vibrant health system is equity. UHC must not simply be universal, but also fair. Many countries purport to offer universal coverage, but they provide inferior services for certain groups such as those living in rural communities. In some countries, to take another example, health laws provide different health benefits depending on the insurance scheme, violating equity. Often the services offered in poor neighborhoods are of lower quality than in high-income communities. Among the varied reasons for inequitable distribution of health services is that skilled health workers are often heavily skewed to working in high-income urban areas, while lacking in poorer, more rural areas. Every person has a right to a roughly equal set of services, with uniformly high quality. Affording certain communities fewer services or lesser quality violates the letter and spirit of UHC. By enacting strong public health laws, governments can allocate services more equitably across populations and geographic areas.

Both the UN and WHO emphasize the importance of affordability. Requiring user fees for health services will render services unaffordable for the poor.^18^ Consequently, poorer populations will delay or avoid seeking healthcare if they are required to pay user fees. Further, accessing services should not lead to impoverishment. In the United States, for example, surprise medical billing has become a major issue, as it often pushes families into bankruptcy.^19^ Governments should provide UHC through pooled, pre-paid funds. Funding for UHC should come from progressive taxation, with governments ensuring that everyone in society, according to their means, pays their fair share of taxes for the public good. Tax avoidance, in other words, can erode funding for, and trust in, the health system.

Health services for all means little if those services are not of uniformly high quality. Laws and regulations, for example, can ensure that pharmaceuticals are safe and effective; physicians are well qualified; hospitals meet certification standards; and health facilities avoid medical errors or hospital-acquired infections. In the search for universal coverage, we often forget the importance of high-quality services, but quality is essential. More than 5 million – and possibly 8 million or more – deaths in low- and middle-income countries in 2015 alone were attributable to poor quality care.^20^

Finally, health systems must be cost effective. No country has an unlimited budget for health services, and governments must balance health services with other important national priorities, such as education, transportation, infrastructure, and social safety nets. Thus, national legislation can appropriately limit guaranteed health services, guided by evidence of what interventions are most effective and how much they cost, and consistent with robust health budgets. Criteria for decisions on what interventions are covered should be transparent. Many countries limit medical spending by negotiating drug prices and/or refusing to cover high-priced services that have relatively low effectiveness compared with other more cost-effective services.

## The Imperative of Robust Financing

National health budgets are primarily important, but many low- and middle-income countries do not have the financial resources needed to ensure high quality health services for all.^21^ The international community should help close the financing gap for UHC. Robust funding for health systems requires two transformations in development assistance for health (DAH). First and foremost, countries must expand their budgets for DAH. For example, while the United States consistently provides more funding for DAH than any other country, high-income European nations far surpass the US’s assistance in per capita and other expenditure measurements.^22^ Many countries, including the Unites States, make contributions far below agreed-upon international targets for development assistance.^23^ Thus, while governments themselves have primary responsibility for funding their health systems, the international community should help close fund deficits through spending a greater proportion of their gross domestic product on international health assistance, and directing that assistance to achieving UHC.

## Good Governance for Health

Even if national health laws adequately address these five core values, there are additional requirements for ensuring healthy populations. Health systems must be well-governed. Good governance requires evidence-based targets, monitoring and measuring outcomes, inclusive participation, transparency, honesty, and accountability. It is impossible to know if health systems are meeting population needs without carefully evaluating outcomes, based on full transparency. Public officials, health workers, and hospitals must be good stewards of health resources. Thus, active measures to combat waste and corruption are essential. And there must be systems of accountability for meeting key health system objectives.

We also need high-quality information, including sub-population data. It is impossible to track health disparities without understanding who is being left behind and whether policies intended to end these disparities are working. The only way to close the health equity gap is to measure health outcomes with granular data, and then act on those data.

Finally, but importantly, governments must abide by the rule of law. If people are subjected to discrimination or marginalization, their health is undermined. If the political and judicial systems are poorly functioning, we cannot achieve health justice. And if civil society freedoms are suppressed, people’s health and safety will be threatened.

While many people think of UHC as a purely scientific, technologic pursuit, in truth, good law and governance are vital for the health and safety of populations everywhere. And law must assure all the conditions needed for good health and well-being, encompassing high-quality healthcare services, public health services, and the social determinants of health. Law, of course, is not the only tool to achieve global health with justice, but it is uniquely powerful.

## Ethical issues

Not applicable.

## Competing interests

Author declares that he has no competing interests.

## Author’s contribution

LOG is the single author of the paper.
